# Rare and common variants in *ERAP1* and *ERAP2* selected for in response to *Yersinia pestis* infection contribute to autoimmune disease including inflammatory bowel disease

**DOI:** 10.1007/s00335-025-10183-3

**Published:** 2025-12-22

**Authors:** Lynn K. Win, Guo Cheng, James J. Ashton, Alex Z. Kadhim, Zachary Green, R. Mark Beattie, Sarah Ennis

**Affiliations:** 1 Human Development and Health, Faculty of Medicine, University Hospital Southampton, Southampton, Hampshire SO16 6YD UK; 2 Southampton General Hospital, NIHR Southampton Biomedical Research Centre, Southampton Centre for Biomedical Research, Mailpoint 218, Tremona Road, Southampton, SO16 6YD UK; 3 Department of Paediatric Gastroenterology, Southampton Children’s Hospital, Southampton, UK

**Keywords:** Autoimmune comorbidity, GenePy, Genetics, Endoplasmic reticulum aminopeptidase (ERAP), Inflammatory bowel disease

## Abstract

Endoplasmic reticulum aminopeptidases (ERAP) facilitate antigen presentation. Variation in *ERAP1* and *ERAP2* genes underwent selection during the Black Death and impact autoimmune disease susceptibility. We assess the burden of functionally impactful variants present in *ERAP1* and *ERAP2* in 2 cohorts of patients diagnosed with inflammatory bowel disease (IBD; Crohn’s disease (CD) or ulcerative colitis (UC)). We analysed the Southampton Genetics of IBD cohort comprising patients diagnosed with IBD, with exome and clinical data on autoimmune comorbidities (n_CD_ = 661, n_UC_ = 330); and a subset of UK Biobank IBD patients and selected controls (n_CD_ = 891, n_UC_ = 1409, n_control_ = 60,075). Common single variants (minor-allele-frequency > 0.05) underwent Fisher’s exact test with permutations (n = 1000), comparing UC versus CD patients in the Southampton cohort. A gene-burden based test of functionally impactful variants (GenePy) was used to examine rare and common variants in both cohorts. Predicted haplotypes were assessed in each cohort and compared with known haplotypes of *ERAP1* and *ERAP2*. In the Southampton cohort, variant-level analysis identified 6 common variants that were nominally significant when testing between UC versus CD (*p* < 0.05; empirical permuted p-value). Gene-level analysis using Mann–Whitney-U identified an altered burden of functionally impactful variants for *ERAP1* in UC versus CD patients (*p* = 0.0073), but not for *ERAP2*. Patients with an isolated diagnosis of UC (*p* = 0.037,θ = 0.457) vs patients with a concurrent diagnosis of UC and any autoimmune diagnoses had an altered burden of functionally impactful variants in *ERAP2*. For the UK Biobank cohort, participants diagnosed with UC versus controls had a significantly altered burden of functionally impactful variants in *ERAP1* (*p* = 0.0004) and *ERAP2* (*p* = 0.0006). Both variant and gene-level analysis indicate a role for *ERAP1* and *ERAP2* in predisposing individuals to developing UC or UC with concurrent autoimmune diagnosis.

**Supplementary Information:**

The online version contains supplementary material available at 10.1007/s00335-025-10183-3.

## Introduction

Endoplasmic reticulum aminopeptidases (ERAP) 1 and 2 are essential proteins involved in the processing of antigens for presentation via the major histocompatibility complex class I (MHC-I) to CD8^+^ T cells (Yao et al. 2019). Insufficiently processed antigens can compromise the stability of the MHC-I complex, leading to reduced expression of MHC-I molecules, which diminishes the immune response and hampers the clearance of infections (Hutchinson et al. 2021). The genes that encode the ERAP proteins, *ERAP1* and *ERAP2,* are located adjacent to each other on 5q15 (Hutchinson et al. 2021; Paladini et al. 2020) and both have been implicated in the development of multiple autoimmune diseases, including Bechet’s disease, birdshot uveitis, ankylosing spondylitis (AS) and inflammatory bowel disease (IBD) (Hutchinson et al. 2021; López de Castro 2018; Yao et al. 2019).

Common functional variants are often associated with autoimmune disease playing a key role in determining ERAP enzymatic activity (Hutchinson et al. 2021; López de Castro 2018; Yao et al. 2019). For instance, AS-associated single nucleotide polymorphism (SNP) rs30187 C > T in *ERAP1* influences the protein’s role in antigen processing. The T allele (allele frequency of 0.346 in non-Finnish Europeans in gnomAD v3, is directly correlated with lower enzyme activity, where the presence of the reference C allele results in a higher-activity ERAP1 protein, leading to exaggerated immune responses to self-antigens and contributing to the development of autoimmune disease. This is likely consequent to the positive selection of high activity ERAP enzymes in the context of the Black Death pandemic, where increased enzyme activity was associated with robust immune response against *Yersinia pestis* bacteria and improved survival (Yao et al. 2019). This vital function of *ERAP* gene alleles during the Black Death may had resulted in a higher prevalence of hypermorphic functional variants in modern European populations.

The prevalence of autoimmune diseases in the UK populations has increased since 2000 (Conrad et al. 2023). A particularly noticeable change is in IBD, in which prevalence has been rising throughout 2000’s to 142.1 per 10,000 adults in the UK affected in 2016 (Pasvol et al. 2020). Incidence of IBD across UK and Europe has been rising and with increasing population size this will result in a higher number of individuals being diagnosed with IBD (Hracs et al. 2025; Nartey et al. 2021).

Genome-wide association studies (GWAS) have previously identified 7 SNPs in the *ERAP* gene region that were associated with IBD. Among these, 3 specifically linked with an isolated diagnosis of CD (Franke et al. 2010; de Lange et al. 2017; Liu et al. 2023) while the remaining 4 exhibiting pleiotropic effects associated with UC, CD or other autoimmune diseases (Ellinghaus et al. 2016; Li et al. 2015). One of the 3 variants associated with increasing susceptibility to developing CD was also identified by Klunk et al. in *ERAP2* (rs2549794) and was found to have the strongest positive selection by the Black Death (Franke et al. 2010; Klunk et al. 2022). However, the impact of this variant on patients with UC has not been explored (Franke et al. 2010). The presence of rs2549794-C allele results in increased production of ERAP2 protein, improving the immune response to *Yersinia pestis* and contributing to the risk of developing Crohn’s disease in contemporary populations. A large case control GWAS study by Liu et al. identified the second variant associated with isolated CD in *ERAP2* (rs6873866) (Liu et al. 2023). This association was not observed in patients with UC. The intronic variant rs6873866 has been shown to regulate gene expression in CD4^+^ regulatory T cells, resulting in the stimulation of type 1 interferon (Bossini-Castillo et al. 2022). This mechanism may play a role in the aetiology of Crohn’s disease by altering the immune response to infections. The third variant associated with CD, rs1363907 was assessed in a group of patients with a diagnosis of either CD or UC (Liu et al. 2015). Specifically, the rs1363907-A allele was found to be associated with CD in European populations. Individuals with 2 copies of the reference rs1363907-G allele were found to have reduced *ERAP2* expression in the liver (Nicoletti et al. 2023). The presence of the alternative A allele in individuals with CD suggests that a higher level of functionally active ERAP2 protein could be contributing to disease, in keeping with the findings of Klunk et al.

While rare genetic variants are believed to contribute to the heritability of complex diseases like IBD (Momozawa and Mizukami 2021), no rare variants in *ERAP1* or *ERAP2* have been specifically linked with the development of CD or UC. GWAS focused on common variants and was unable to investigate rare variants that may exert stronger effects on disease susceptibility (Young 2019). Whole exome sequencing (WES) provides a more comprehensive approach than GWAS by capturing all variation across coding regions of the genome, identifying variants resulting in altered protein expression, structure or functionality, and enabling the identification of rare variants, with clinically impactful effect sizes (Ross et al. 2020). Leveraging WES data for disease-association tests using a gene-based burden testing approach is superior to GWAS or single variant testing in multiple ways (Guo et al. 2018). By aggregating the effects of rare variants across a gene in conjunction with common variants, gene-burden based tests improves the power to detect signals, reduces the need for multiple test corrections and facilitates comparisons between different groups (Lee et al. 2012; Mossotto et al. 2019).

GenePy, a gene-burden based test, can assess rare and common variations at a cohort and gene level (Mossotto et al. 2019). GenePy scores are generated on a per individual, per gene basis using population allele frequency, individual zygosity determined through variant calling, and a user-defined deleteriousness metric to assess functional impact. We used Combined Annotation Dependent Depletion (CADD) deleteriousness score (v1.6) as this provides predictions for all variants (Rentzsch et al. 2019).

This study employed both single variant and whole gene-based approaches to assess the burden of deleterious variants *ERAP1* and *ERAP2* in the UK Biobank and a local cohort of IBD patients with WES and deep clinical phenotype data. We evaluated the burden of variants in the *ERAP* genes between IBD patients versus controls in the UK Biobank cohort. In the Southampton IBD disease only cohort, we assessed the frequency and distribution of variants firstly between all patients diagnosed with CD and UC, and secondly between patients with an isolated IBD diagnoses versus those more complex IBD patients with additional autoimmune diagnoses. This allowed us to review the aggregated effect rare variants between individuals with and without IBD, between IBD subtypes and those with additional autoimmune diagnoses.

## Methods

### The Southampton IBD and UK biobank cohorts

The primary cohort for this analysis was the patient-only Southampton Genetics of IBD Study, approved by the Southampton and Southwest Hampshire Research Ethics Committee (09/H0504/125). Adult and paediatric patients have been recruited since 2010 from Southampton Children’s Hospital and University Hospital Southampton. Diagnoses of UC and CD were confirmed using the Porto Criteria for paediatric patients and the Montreal criteria for adults (Satsangi et al. 2006). All patients had longitudinal deep phenotype data and underwent WES.

The second cohort examined was UK Biobank Phase 2 data (UK Biobank project 72,911), which included ~ 200,000 participants with deep phenotype and WES data (UKBiobank 2023) generated using the IDT xGen Exome Research Panel v1.0 capture kit (Halldorsson et al. 2022). Gene-centric case–control association tests were conducted for UC and CD separately within this dataset. The analysis focused on Caucasian participants across 3 groups, with patients defined according to their diagnoses. Individuals with CD and UC were identified using ICD-10 coding. Controls were selected as participants with no recorded history of immune-related or digestive disease (Sudlow et al. 2015).

Potential confounding factors that may have impacted our analysis were accounted for by using sample-swapping permutations. We focus on patients and controls of European ancestry only as they represent the majority of both cohorts.

### Clinical phenotyping

Clinical data, including autoimmune comorbidities, were extracted from electronic patient records. Given that *ERAP* genes have been associated with multiple autoimmune diseases, we focused on identifying IBD patients with or without an additional diagnosis of another autoimmune disease. The list of common autoimmune diseases considered was based on the most common autoimmune diseases present in UK Biobank (Bycroft et al. 2018) those listed in the Connect Immune Research network (British Society for Immunology 2024) and Medline (A.D.A.M. Medical Encyclopedia 2024). The full list is presented in supplementary Table 1.

Using autoimmune comorbidity data from the clinical records, the Southampton IBD cohort was subdivided into 4 groups: patients with an isolated diagnosis of CD, those with the diagnosis of CD and at least 1 additional autoimmune diagnosis, patients with an isolated diagnosis of UD and those with a diagnosis of UC and at least 1 additional autoimmune diagnosis. A comparison of each subtype with and without additional autoimmune diseases was conducted using Mann–Whitney U on the whole gene burden scores for *ERAP1* and *ERAP2.* The distribution of GenePy scores for each gene were visualised in R version 4.3.1 (Firth et al. 2009) using ggplot2 (Wickham et al. 2019).

### Processing and quality control of whole exome-sequencing data for the Southampton cohort

WES data was generated from germline DNA, using Agilent SureSelect All Exon v5 and v6 exome capture kits (Andreoletti et al. 2015). Alignment to the human reference genome (GRCh38 assembly with decoy human leukocyte antigen regions) was performed using BWA-mem aligner v.0.7.17 (Li 2013). Individual samples had duplicate reads flagged before undergoing base quality score recalibration. Variant calling using HaplotypeCaller (GATK 4.2.2.0) was conducted on the region defined by the union of the 2 capture kits with 150 bp padding (Van der Auwera and O’Connor 2020). All samples were joint called with GenomicsDB and GenotypeGVCFs (GATK 4.2.2.0), generating a whole cohort VCF file. To select variants across the *ERAP* genes: 1) exonic targets falling within the GENCODE 45 defined gene regions for *ERAP1* and *ERAP2* were identified from the capture kit BED files; these BED file targets were then padded by 100 bp (in accordance with manufacturers guidelines); only variants falling within the intersection of these padded targeted regions from either SureSelect v5 or V6 were retained. These steps ensured minimal variability of capture and coverage between samples.

Quality control (QC) was applied soft filtration using variant quality score recalibration to flag potential false positive reads (Van der Auwera and O’Connor 2020) followed by hard filtration, removing genotypes with genotype quality < 20, sequencing depth < 8, and retaining only variants with missingness in ≤ 10% of the cohort (Carson et al. 2014).

**Fig. 1:**
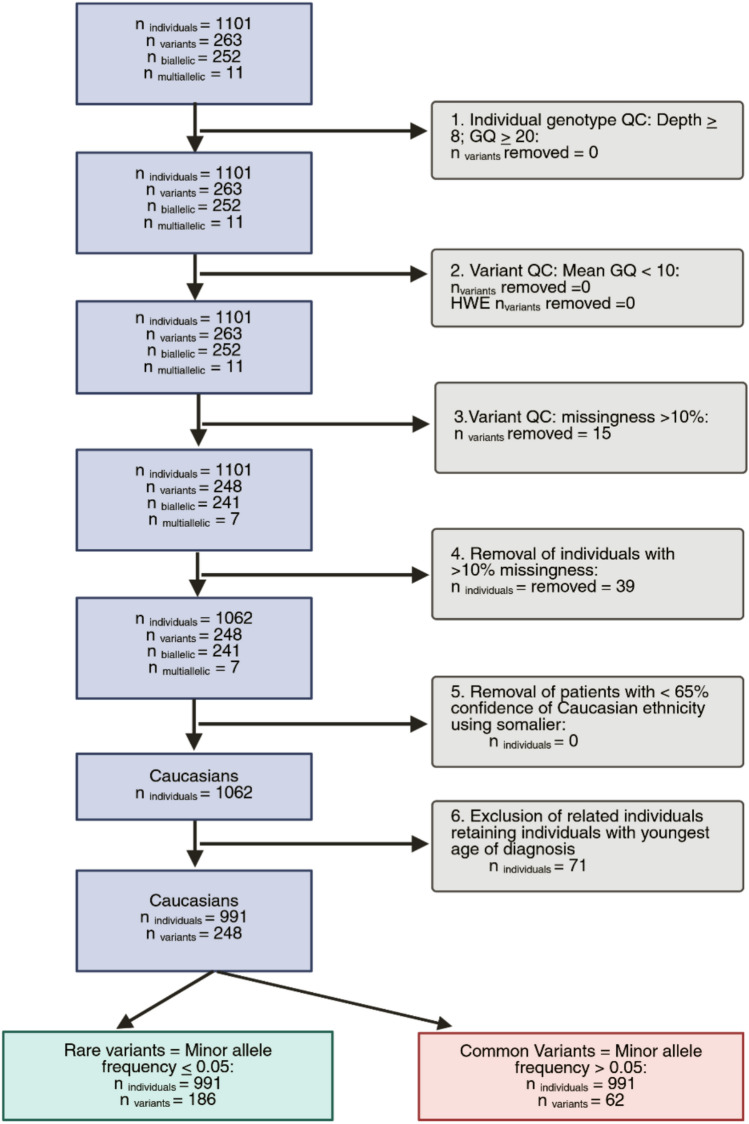
Workflow detailing the process of selecting the interval, samples, and variants in the Southampton IBD cohort. An interval including the coordinates of *ERAP1* (ENST00000443439.7) and *ERAP2* (ENST00000437043.8) was selected. As per Carson et al., individual genotype QC and variant QC was then carried out.^37^ Variants that were missing in > 10% of the population were removed as this may represent poor genotyping at that position. Individuals missing > 10% of the target region were removed as this may represent poor DNA quality. Somalier was used to assign genetically derived ethnicity. A cutoff of > 65% likelihood was used to derive ethnicity. For self-reported related individuals, the individual with the youngest age at diagnosis was retained for the analysis. Genes were split into rare and common variants based on the minor allele frequency of 0.05 in this callset. Common variants (allele frequency > 0.05) underwent Fisher’s exact test with sample swapping permutation (n = 1000)

For individual patient samples, those missing > 10% sequence data were excluded. Somalier was used to predict and retain samples of Caucasian ethnicity, as per the developer’s recommendations a < 0.65 confidence threshold was applied to define ethnicity (Pedersen et al. 2020). Where relatives were identified, only the youngest diagnosed individual in each self-reported family group was retained (Fig. 1). A common SNP panel was used as an orthogonal method to confirm data handling retained sample identity (Pengelly et al. 2013). Hardy–Weinberg calculations were used to scrutinise population allele frequencies and assess where these deviated from expected frequencies predicted using Hardy–Weinberg equilibrium (Rentzsch et al. 2019).

Variant function was annotated using the Ensembl Variant Effect Predictor (VEP) version 1.09 (Hunt et al. 2022) and predicted functional impact was evaluated based on CADD v1.6. CADD is an *in-silico* tool that predicts the functional consequence of a variant using a framework that integrates multiple annotations into 1 metric and contrasts variants that survived natural selection with simulated mutations (Rentzsch et al. 2019). CADD scores can be generated for SNPs, insertions/deletions and multi-nucleotide substitutions. These metrics are used to generate raw and scaled (CADD_PHRED_) scaled scores. The PHRED scales CADD scores represent a probability that the variant has a deleterious effect, with higher scores associated with an increased chance of deleteriousness. A CADD_PHRED_ score > 20, equating to a raw score in the top 1% of all scores. A threshold of CADD_PHRED_ > 15 was used to filter likely functionally impactful variants. Allele frequency in non-Finnish Europeans was assigned using gnomADg v3.1.2 and CADD_PHRED_ scores are presented in supplementary tables 2 (common variants) and 3 (rare variants).

### Single variant annotation and association testing

After QC, all remaining variants in the target region encompassing *ERAP1* and *ERAP2*: chr5:96,774,484 – 96,919,703 (GRCh38 and as all genomic coordinates shown in this paper) were retrieved in the Southampton IBD cohort (Kent et al. 2002). Called variants were defined as common or rare using a threshold of minor allele frequency (MAF > 0.05). Statistical tests assessing the altered frequency of common variants within UC versus CD subgroups was conducted on the Southampton patient-only IBD cohort. Fisher’s exact test with sample-swapping permutations (n = 1,000) was applied and the test executed using PLINK 1.9 (Purcell et al. 2007). Variants with (*p* < 0.05) were extracted. Linkage disequilibrium (LD) between SNPs with significant empirical permuted p-values (EMP) and the 3 variants that were associated with CD was estimated using the correlation coefficient (r^2^) based on the European population in the 1000 Genome project using LDpop (Alexander and Machiela 2020).

Common and rare variants were annotated with by type (e.g. missense, splice), allele frequency in non-Finnish Europeans (gnomADg v3.1.2) and CADD_PHRED_ (CADD v1.6) score (supplementary tables 2 and 3 respectively). Rare variants, not meeting statistical criteria for single variant-based tests, were analysed for their aggregated effect through gene-burden based tests.

### Individual haplotype prediction

Ombrello et al. described 10 haplotypes in *ERAP1* identified by 9 tagging SNPs (Supplementary Fig. 1) and associated with increased, moderate or decreased *ERAP1* activity. Kuiper and Raja identified 10 haplotypes for *ERAP2*, identified by 11 tagging SNPs associated with normal or decreased *ERAP2* activity. Not all tagging SNPs for these previously reported functional haplotypes were available from the exomes sequencing data used in this study, but those captured and passing quality control were used to generate haplotypes and assess their frequencies across both cohorts. Common tagging SNPs (MAF > 0.05) contributing to these haplotypes were tested for association between haplotypes, comparing patients with UC vs CD in the Southampton cohort. In UK Biobank cohort, controls were compared with CD and UC patient groups and between patients with UC and CD.

### Gene-based burden tests on functional variants

Gene-based burden tests focused on variants predicted by CADD to confer functional consequences (CADD_PHRED_ ≥ 15) in the coding and adjacent regions of the *ERAP1* (chr5:96,774,459–96,807,960) and *ERAP2* (chr5:96,876,475–96,919,728). Individual genes were defined using GENCODE version 43 with additional padding of 25 base pairs (bp) either side of coding regions to include variants most likely to impact splicing (Pujar et al. 2018).

GenePy was used to integrate the impact of common and rare variation for testing across entire genes in a single test. GenePy is a gene-level scoring system that integrates information on zygosity, population frequency, and functional deleteriousness for all observed variants in any given gene per individual. Whole gene GenePy scores are intuitive in that individuals with highest scores carrying the highest burden of functionally impactful variants. This quantitative score facilitates relative comparisons at the individual level or statistical testing between groups.

In the Southampton IBD patient-only cohort, Mann–Whitney U was used to compare the GenePy scores between patients diagnosed with IBD subtype (i.e. CD compared to UC). This cohort was subdivided into those with a diagnosis of isolated CD (i.e. no other concurrent autoimmune diagnoses) vs those with CD and ≥ 1 autoimmune disease, those with isolated UC and those with CD and ≥ 1 autoimmune disease and their GenePy scores compared.

In the UK Biobank cohort, a comparison of GenePy scores using Mann–Whitney U was conducted comparing control participants against individuals diagnosed with CD or UC. GenePy scores were generated for *ERAP1* and *ERAP2* for every individual. However large scale GWAS indicate that *ERAP1* and *ERAP2* are not common major drivers of autoimmune disease. We hypothesise that only a small but unknown percentage of patients have functionally impactful variation in *ERAP* genes which results in altered autoimmune disease susceptibility. GenePy score distributions are non-normal and are specific to each gene. Both gene size and conservation can affect the range and distribution of GenePy scores generated for any given gene. As such treating the GenePy score as a continuous variable would not be statistically or biologically appropriate. Previous publications using GenePy (Sunny et al. 2025) identify that GenePy is powered and sensitive to detect genetic drivers, but that non-parametric testing of individuals with the most extreme scores is effective to identify biologically known and new signals. SKAT-O and other traditional methods do not have the flexibility to reflect the pathology of genetic diseases where pathogenic variants only affect a small percentage of patients.

GenePy scores for *ERAP1* and *ERAP2* were ranked in descending order for the CD, UC and control groups separately. We used a range of 1–10% of IBD cases in intervals of 1%, 2.5%, 5% 7.5% and 10% to tune our calculations as to what percentage of individuals had *ERAP1* and *ERAP2* as an important driver of their IBD. Testing over this interval resulted in the choice to use a cut off of 7.5%. Theta (θ) was used to assess the directionality of effect (Newcombe 2006). This statistic quantifies the degree of difference between the groups where a θ value of 0.5 suggested no difference between groups, a value > 0.5 indicates that the cases are more likely to have higher values than the controls and a value of < 0.5 indicated the opposite.

## Results

### The Southampton IBD cohort: demographics, disease subtypes and autoimmune comorbidities

Following QC, the Southampton cohort comprised 991 unrelated Caucasian patients (n_CD_ = 661, n_UC_ = 330) (Fig. 1). Table 1 details the Southampton IBD cohort by subtype, autoimmune disease status, gender, median follow up time, and age at diagnosis of IBD. There was an expected excess of males in CD patients (n = 366; 55.37%) compared to UC patients (n = 163; 49.39%) (*Χ*^2^ test *p* = 0.075). Median follow-up time for paediatric patients diagnosed with CD or UC was 9.5 years. Patients diagnosed with adult-onset CD had a median follow up time of 17.9 years and 10.7 years for those diagnosed with UC.

**Table 1:** Summary of Southampton IBD cohort demographics after QC, removal of non-Caucasian patients and related individuals

	*Onset of disease*	*Total*	*Median age at diagnosis (IQR)*	*Male*	*Female*	*Median time of follow up (IQR)*	*Number of patients with* ≥ *1 autoimmune comorbidity (%)*
*Crohn’s Disease (CD)*	Paediatric	392	13.0 (10.7 -14.8)	244	148	9.5 (4.8—12.6)	30 (7.7)
	Adult	269	31.0 (23.0- 42.0)	122	147	17.9 (12.1—23.6)	50 (18.6)
	All	661	15.5 (12.2—26.0)	366	295	13.2 (9.3—20.9)	80 (12.1)
*Ulcerative Colitis (UC)*	Paediatric	179	13.2 (10.0—14.9)	87	92	9.5 (3.7—12.5)	21(11.7)
	Adult	151	34.0 (26.0—46.5)	76	75	10.7 (8.0—13.0)	18(11.9)
	All	330	15.7 (13.1 -31.8)	163	167	9.8 (6.9—12.6)	39(11.8)

*Abbreviation: IQR* Interquartile range

**Fig. 2:**
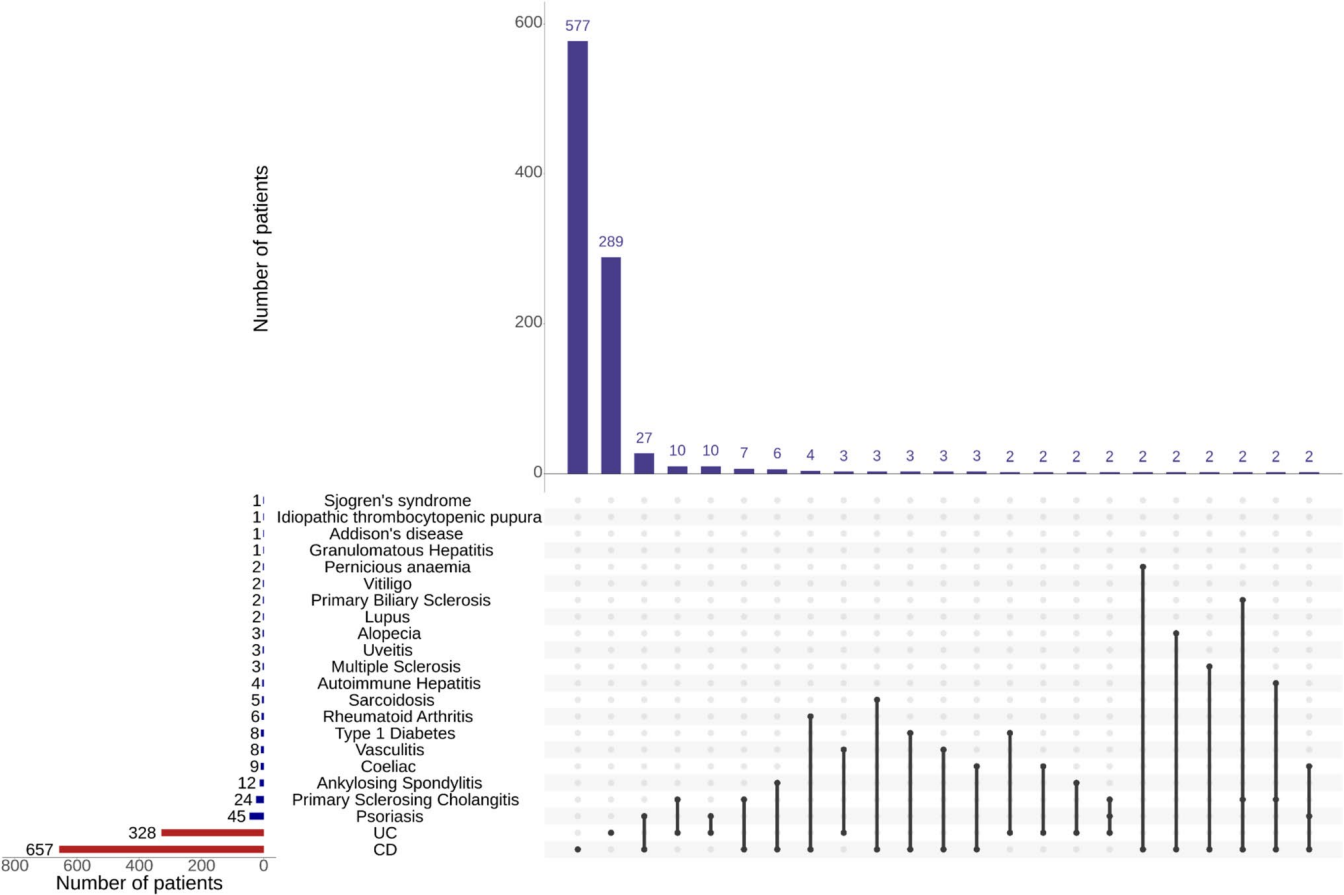
The distribution of IBD subtype and concurrence of autoimmune comorbidities in the Southampton IBD cohort. A total of 22 diseases were plotted and are listed on the left side of the matrix. The total number of patients diagnosed with each autoimmune disease are shown in the bar chart to the left. IBD subtypes are highlighted in orange (CD Crohn’s disease, UC Ulcerative colitis). The matrix in the centre shows the intersection of IBD and concurrent autoimmune disease and the bar chart above the matrix the number of individuals within the Southampton cohort who have a combination of those diseases. Intersections containing only one patient have been excluded from this plot

In the Southampton IBD cohort, 119 patients had a concurrent diagnosis of 1 or more additional autoimmune disease(s). A higher proportion of adults were diagnosed with CD and at least 1 other autoimmune disease (18.6%) compared to just 7.7% of patients diagnosed in the paediatric clinic—possibly reflecting increased duration of opportunity to be diagnosed with an additional disease in the adult group. However, for UC the proportion of patients having an additional autoimmune disease was consistent across both adult-onset and paediatric-onset disease. The distribution of autoimmune disease types and their frequencies within the cohort is presented in Fig. 2.

### Comparison of common variants in ERAP genes between IBD subtypes in the Southampton IBD cohort

Following QC, a total of 248 variants were identified across the region covering *ERAP1, ERAP2* genes, of which 62 were common (MAF > 0.05) and underwent Fisher’s exact test with sample-swapping permutations (n = 1,000), comparing frequency of variants between patients with UC and CD. Allele frequencies observed in the Southampton cohort are largely consistent with those observed in the gnomAD genome data for non-Finnish Europeans. Of the 62 common variants eligible for single variant analysis, 6 showed nominally significant differences comparing UC and CD (p_EMP_<0.05) but none withstood FDR correction (Table 2). Of these, 5 were in the *ERAP1* gene region (Table 2), including 2 missense, 2 synonymous and 1 intronic variants. The missense variants had CADD_PHRED_ scores that predicted a high likelihood of having a functionally significant impact. These 2 missense variants contribute to functionally important haplotypes of *ERAP1* (Ombrello et al. 2015). All the 6 nominally significant variants identified as differentially distributed between patients with UC and CD in the Southampton cohort appear to be novel. None of these variants have been identified in GWAS. Additionally, none were in strong LD with previously identified SNPs from the literature (supplementary Table 4).

**Table 2:** Fisher’s exact test of *ERAP1* & *ERAP2* in CD versus UC of the Southampton IBD cohort

											Fisher’s exact test (UC versus CD)					Permuted *p* values	
Gene	Chr	Position	rsID	Ref	Alt	Consequence	gnomADg NFE alternative allele	CD alternative allele frequency	UC alternative allele frequency	CADD Phred (v1.6)	p value	OR	SE	Lower 95% CI	Upper 95% CI	Empirical	Corrected empirical
*ERAP1*	5	96,780,408	rs34765952	ATT	AT	synonymous	0.217	0.194	0.246	0.45	0.021	0.738	0.129	0.573	0.951	0.019	0.316
***ERAP1***	**5**	**96,783,162**	**rs17482078**	**C**	**T**	**missense**	**0.209**	**0.188**	**0.230**	**23.100**	**0.032**	**0.775**	**0.119**	**0.613**	**0.979**	**0.040**	**0.446**
*ERAP1*	5	96,785,820	rs469783	T	C	synonymous	0.434	0.462	0.414	8.240	0.043	1.217	0.096	1.009	1.469	0.045	0.534
***ERAP1***	**5**	**96,793,832**	**rs2287987**	**T**	**C**	**missense**	**0.207**	**0.190**	**0.236**	**14.830**	**0.021**	**0.761**	**0.119**	**0.603**	**0.960**	**0.025**	**0.32**
*ERAP1*	5	96,803,363	rs10062964	C	T	Intronic	0.207	0.189	0.235	2.298	0.021	0.760	0.119	0.602	0.958	0.026	0.32
*ERAP2*	5	96,879,976	rs41506651	C	T	synonymous	0.099	0.120	0.087	3.410	0.025	1.427	0.155	1.054	1.932	0.016	0.35

Abbreviations: *CD* Crohn’s disease, *UC*  Ulcerative Colitis, *rsID*  Reference SNP cluster ID, *Ref*  Reference, *Alt* Alternative, *gnomADg*  NFE gnomAD genomes, Non-Finnish European, *OR*  Odds ratio, *SE*  Standard error, *CI*  Confidence interval. Values in bold represent variants that are associated with known European haplotypes as identified by Ombrello et al

### Predicted haplotypes and their functional impact in the Southampton IBD and UK biobank

Ombrello et al. and Kuiper and Raja previously identified functionally impactful haplotypes. While not all tagging SNPs from these haplotypes were present within our exome source data, we used those tagging SNPs that were present in the Southampton and UK Biobank exome data to assess haplotype frequency in those cohorts. Across both cohorts 8 haplotypes (minimum haplotype frequency = 0.01) were identified in *ERAP1*, and 4 haplotypes were identified in *ERAP2*.

Haplotype association with disease subtypes and controls was carried out using common variants from the tagging SNPs present in our cohorts. In *ERAP1*, 8 variants passing QC and MAF-based filtration were used to generate haplotypes. In *ERAP2*, only 3 SNPs passed MAF-based filtration (rs2549782, rs2248374, rs17408150) and their distribution was compared between groups in both cohorts. We observed no significant association with haplotypes of *ERAP2* between any of the subgroups. For *ERAP1*, haplotype A identified by our group, that partially mapped to Ombrello haplotype 4, was differentially distributed between CD vs controls (*p* = 0.005, OR = 1.207) and CD vs UC (*p* = 0.009, OR = 1.256) in the UK Biobank (supplementary table 6). Haplotype F, that had no corresponding Ombrello haplotype was differentially distributed between CD and UC (*p* = 0.043, OR 0.784) but not in the Southampton IBD cohort. Furthermore, we identified haplotype E that did not map to and Ombrello haplotype that was differentially distributed between patients with UC and CD (*p* = 0.029 OR = 0.770) in the Southampton cohort that was found at a modest but significantly higher rate in patients with CD (supplementary table 6).

### Distribution of rare variation across the Southampton IBD cohort

**Fig. 3:**
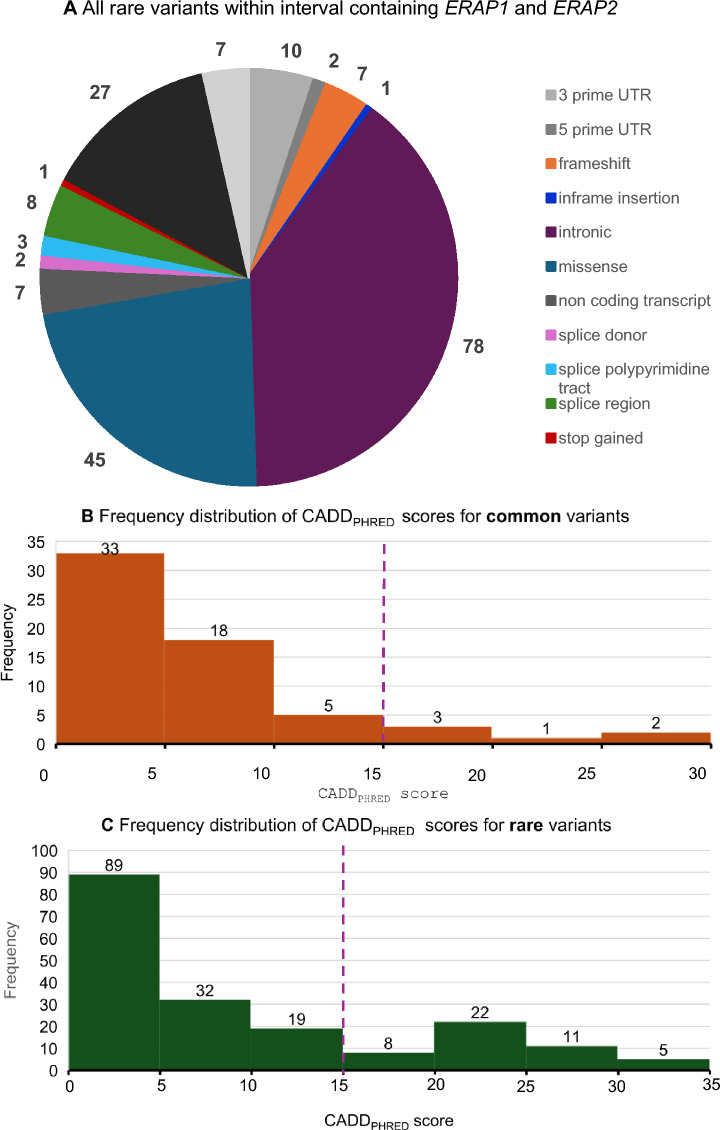
High quality rare variant classification (n = 186) within the Southampton IBD cohort (n = 991). CADD is a deleteriousness metric. A threshold of CADD_PHRED_ > 15 was used to filter likely functionally impactful variants. **A.** Genomic region encompassing *ERAP1* and *ERAP2.* **B.** the frequency distribution of CADD_PHRED_ scores for common variants **C.** the frequency of rare variants and their CADD_PHRED_ scores. The purple dashed line represents a CADD_PHRED_ score of 15, with scores above 15 representing a greater chance of being functionally significant. UTR, untranslated region

Amongst the 991 patients in the Southampton IBD cohort, 186 rare variants (MAF < 0.05) were identified. Of these, 88 variants fell within *ERAP1*, 98 in *ERAP2.* These 186 variants were categorised by variant type, with the most frequent classed as intronic variants (41.9%; n_variants_ = 78), missense variants (24.2%; n_variants_ = 45) and synonymous variants (14.5%; n_variants_ = 27). The remaining < 20% of variants included splice, frameshift, untranslated region (UTR) and non-coding transcript variants (Fig. 3A). A minority of variants (6.5%; n_variants_ = 12) had multiple classifications. Of the set of unique variants called across the patient cohort, a higher proportion of the rare variants (24.7%; n_variants_ = 46) had a CADD_PHRED_ scores exceeding the threshold of 15 that is generally taken to indicate functional impact, compared to just 9.7% (n_variants_ = 6) amongst the 62 unique common variants identified (Fig. 3B and C). Rare variants are more likely to have an increased predicted functional effect and effect size, resulting in more severe phenotypes (Gorlov et al. 2011). These data argue for the importance of assessing rare variation, that can be collectively common with the potential to impart greater function impact to genes and their protein products.

### Association of ERAP genes with IBD subtypes through gene burden-based tests

While rare variants are not powered for single SNP association testing, their contribution to disease was assessed using the GenePy gene burden-based testing for *ERAP1* and *ERAP2*. These variants are detailed in supplementary Table 3.

**Fig. 4:**
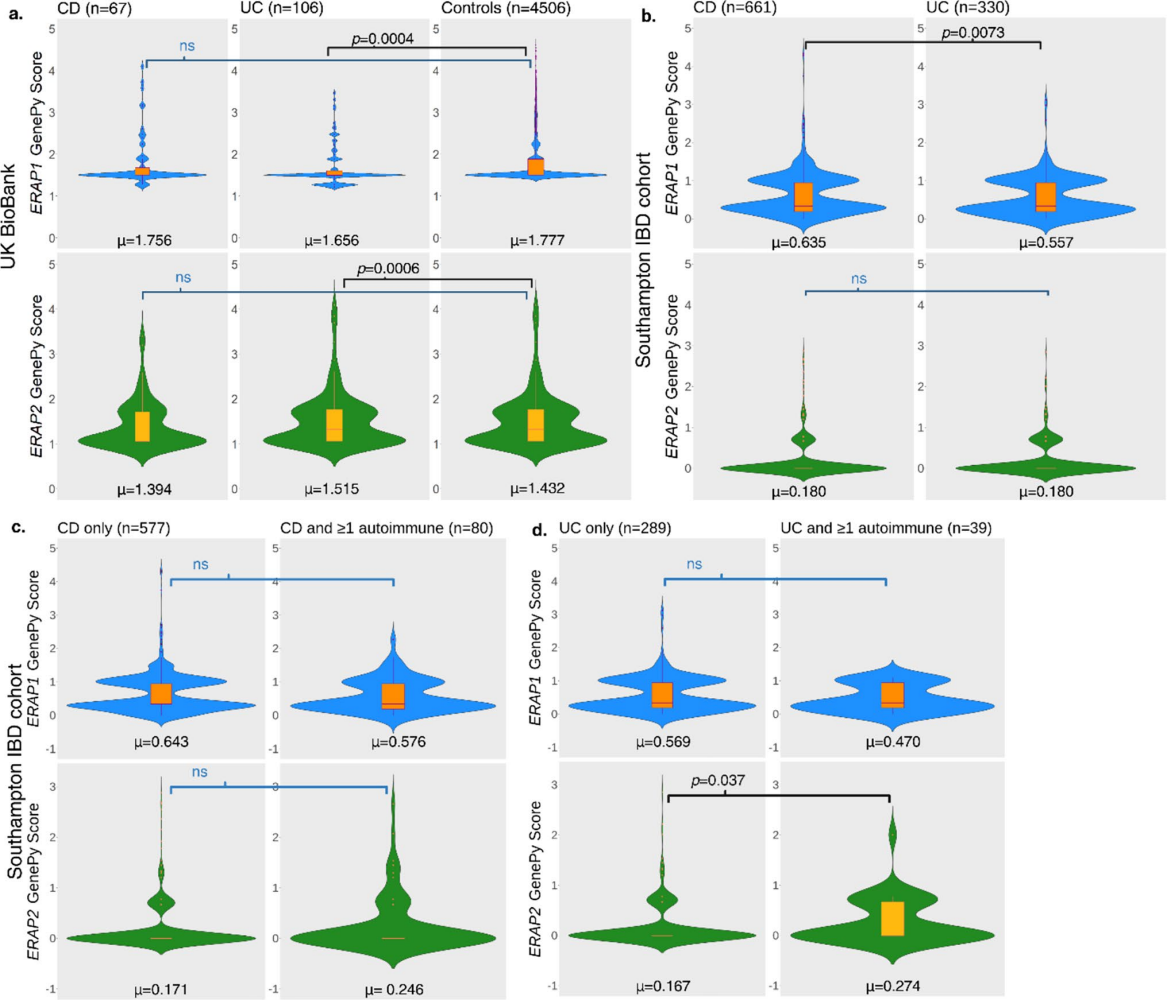
Distribution of GenePy scores for *ERAP1* (blue) and *ERAP2* (green) in and the median and interquartile range (orange): **a.** comparing the top ranked 7.5% of UK Biobank cases vs controls (n_CD_ = 67, n_UC_ = 106, n_controls_ = 4506) **b.** Southampton IBD cohort comparing scores for all CD patients against all UC patients **c.** Southampton IBD cohort comparing patients diagnosed with isolated CD those with CD and at least one additional autoimmune diagnosis. **d.** Southampton IBD cohort comparing patients diagnosed with isolated UC those with UC and at least one additional autoimmune diagnosis. All significant *p* values are shown. ns = non-significant

In the UK Biobank cohort, GenePy scores were ranked in descending order for CD, UC, and control groups and the highest 7.5% of GenePy scores were taken from each of the groups reducing the number of participants to CD (n = 67), UC (n = 106) and controls (n = 4506) and compared. Comparison of the top ranked 1%, 2.5%, 5% and 10% GenePy scores of affected and unaffected participants did not identify a significant difference in their distribution. No significant differences in GenePy scores were observed between CD patients and controls for either *ERAP1* or *ERAP2*. However, GenePy-based analysis revealed significant differences between UC patients and controls for *ERAP1* indicating that participants diagnosed with UC have an altered burden of variation with a predicted functional effect compared to controls (*p* = 0.0004, θ = 0.4110). This θ value suggests that controls have a higher burden of functionally impactful variants in *ERAP1*. Similarly, *ERAP2* GenePy scores indicated a significantly different burden of variants with a predicted functional effect in individuals diagnosed with UC compared to controls (*p* = 0.0006, θ = 0.5941) (Fig. 4A).

It should be noted that reliable interpretation of values of θ greater than or less than 0.5 is compromised by lack of data reliably determining the true direction of effect. This is especially the case for rare missense variants contributing to whole genes scores as these may confer either gain or loss of function but experimental data evidencing this is frequently lacking. Although bi-directional effects have been established for many disease genes, there is no current reliable method to estimate direction of effect for rare missense variants. The presence of impactful variants in controls may lead to reduced ERAP1 activity and reduced inflammation (Reeves and James 2018). Furthermore, because ERAP1 and ERAP2 also function together to trim ~ 30% of peptides it is possible that variation in one gene may be confounded or compensated by variation in the other. Experimental data are essential to fully elucidate the combined impact of genetic changes across both genes.

As the Southampton cohort comprised only patients, no ranking of GenePy scores was required and the whole range of GenePy scores were used when comparing CD and UC patients. Consistent with the UK Biobank analysis, an altered burden of variation predicted to have functional impact was observed for UC patients in *ERAP1* (*p* = 0.0073, θ = 0.3635). No statistically significant difference was observed in GenePy scores for *ERAP2* in this smaller cohort.

### Assessment of association with autoimmune comorbidities

The Southampton cohort was subdivided into 4 categories: patients diagnosed with isolated CD (n = 577), those diagnosed with isolated UC (n = 289), patients with a diagnosis of CD and ≥ 1 autoimmune disease (n = 80) and those diagnosed with UC and ≥ 1 autoimmune disease (n = 39). Mann–Whitney U tests were conducted to compare GenePy scores for *ERAP1* and *ERAP2* between those with isolated CD and CD and ≥ 1 autoimmune disease diagnosis and those with isolated UC and UC and ≥ 1 autoimmune disease diagnosis (Fig. 4C). We observed no significantly altered burden of functionally impactful variation across these groups for *ERAP1* (CD vs CD and ≥ 1 autoimmune disease diagnosis *p* = 0.376, UC vs UC and ≥ 1 autoimmune disease diagnosis *p* = 0.369*)*. However, in *ERAP2* the distribution of GenePy scores of those with concurrent diagnosis of UC and multiple autoimmune diseases harboured significantly inflated GenePy score (*p* = 0.037) compared to those with those with isolated UC (Fig. 4). This was not seen in the comparison of those with CD vs CD and ≥ 1 autoimmune disease diagnosis (*p* = 0.248).

## Discussion

This study identified statistically significant differences in the burden of functionally impactful variation that are persistent in contemporary cohorts of patients diagnosed with IBD subtypes. Examining the Southampton patient-only IBD cohort, we identified altered carriage of functional variation for *ERAP1* but not *ERAP2* comparing disease subtypes*.* When comparing either subtype against a control group in the UK Biobank cohort, we observed that this difference was most evident in the ulcerative colitis patient group for both *ERAP* genes, whereas the Crohn’s disease subgroup did not differ significantly from controls. In the deeply phenotyped Southampton cohort, individuals with multiple autoimmune diseases had a significant difference in the distribution of functional variants in *ERAP2*. Collectively, these data add to the growing evidence of altered antigen presentation having at modifying effect in current patient cohorts diagnosed with effects most prominent in patients with ulcerative colitis or multiple autoimmune diagnoses.

We identified IBD-associated SNPs in this region using from multiple sources including OpenTargets (Ochoa et al. 2020) and GWAS catalogue (v1.03) (Sollis et al. 2023). These variants had their frequency and distribution in the Southampton IBD cohort assessed. All variants in *ERAP1* and *ERAP2* significantly associated with IBD, UC or CD with were included from both sources and are listed in supplementary Table 5. Variants known to be associated with IBD were not present in our cohort or failed QC. These variants were assessed in the Southampton IBD cohort using LD r^2^ scores. While none of the 3 variants associated with CD from previous GWAS studies were in LD with the variants identified by Fisher’s exact test in the Southampton IBD cohort, they were in strong LD with each other. As these 3 variants are being inherited together, it suggests epistatic interactions between them could be resulting in the development of CD. Existing functional evidence suggests that this could be through the production of full length functional ERAP2 protein resulting in an enhanced response to infection and autoimmunity, or through the regulation of gene expression in CD4 + T cells, resulting in immune dysregulation. Further functional studies to understand the interaction of these variants would help clarify how their interplay contributes to CD. Additionally, the effect of rs2549794 should be explored in UC.

Our single variant testing of common variants in the Southampton IBD cohort identified 6 novel variants, 5 in *ERAP1* and 1 in *ERAP2* not previously associated with risk of developing subtypes of IBD. While only 2 were likely functionally impactful based on their CADD_PHRED_ score, it appears that accumulated effects of variants in *ERAP1* results in an overall change in protein function (Reeves and James 2018). The accumulated effects of these variants may affect which subtype of IBD a patient presents with. Two common *ERAP1* SNPs achieving empirical permuted p < 0.05 significance. These had been found by Ombrello et al. in a study examining *ERAP1* haplotypes in European patients with rheumatic disease (Ombrello et al. 2015). In our IBD cohort, the minor allele at each of these SNPs was found to confer a reduced chance of being diagnosed with UC (rs17482078-T OR = 0.775, confidence interval (CI) [0.613–0.979], and rs2287987-C OR = 0.761, CI [0.603–0.960]).

While CADD represents one of the most utilised metrics indicating the functional impact in genetic changes, the score does not convey the direction of effect any functional change may impart. Interpretation is even more difficult in genes that may be subject to balancing selection where deleteriousness is dependent on environmental circumstances or exposure. Analogies can be made with *G6PD*, a gene where dysfunctional variation confers a selective advantage in the presence of *plasmodium falciparum*, but otherwise increases risk of haemolytic anaemia (Mbanefo et al. 2017). But such comparisons overlook the impact of time and the contemporary absence of *Yersinia pestis* as an environmental threat.

Rare variants—unsuited to traditional single variant analysis—had their aggregated effect in IBD and its subtypes assessed using gene burden-based testing. Although individually rare events, rare variants represent the majority class (75%) of unique genetic changes detected through exome sequencing in the Southampton IBD cohort. Their impact is further confounded because as a group, rare variants are enriched for genetic changes predicted to confer functional impact. This is the first study of the aggregated effect of functional variants in *ERAP1* and *ERAP2* in IBD. Using GenePy, we were able to identify differences between the burden of variants of any frequency, predicted to have functional impact between healthy individuals and those with IBD and between disease subtypes, particularly for those diagnosed with UC.

Variation leading to altered function of ERAP1 and ERAP2 can impact the normal functioning of CD8 + T cells and MHC-I presentation (López de Castro 2018). Altered antigen trimming by ERAP1 and ERAP2 results in suboptimal antigen length presentation by MHC-I complexes to CD8 + T cells. CD8 + T Cells are critical for the functioning of the immune system (Koh et al. 2023). Naïve CD8 cells are stimulated by antigens presented by MHC-I complex which causes differentiation to CD8 + cytotoxic cells allowing them to eliminate malignant or infected cells. Suboptimal antigens generated by altered ERAP1 and ERAP2 could trigger responses to commensal instead of pathogenic bacteria resulting in a chronically inflamed state or auto-antigen presentation resulting in autoimmunity (He et al. 2024). An alternative potential mechanism revolves around altered peptide lengths from abnormal ERAP processing of proteins, leading to reduced efficiency of an anti-microbial response. We hypothesise that this may result in insufficient clearance of pathogenic variants resulting in a chronic inflammatory state in the colon. This hypoimmune response is a common factor in multiple genetic causes of IBD (NOD2 deficiency, XIAP deficiency, chronic granulomatous disease-related colitis etc.) (Pedersen et al. 2014).

ERAP dysregulation could preferentially explain the development of UC rather than CD due to several factors. UC is characterised by mucosal ulceration and inflammation, beginning in the rectum and extending continuously into the proximal colon (Zou et al. 2021). A breakdown in microbiome homeostasis and inappropriate inflammatory responses to normal bacterial interactions may represent a larger part of pathogenesis of UC due to its restriction to the colon rather than in CD, which is spread more diffusely throughout the digestive tract. As the microbiome interfaces with the colon at the epithelial level, altered ERAP1 function would more likely result in UC rather than CD which affects the whole thickness of the intestinal tract. 

We identified common haplotypes from the literature in both cohorts to aid with assessing their functional impacts. Haplotype prediction in both cohorts identified the presence of 3 of the Ombrello *ERAP1* haplotypes in both cohorts. One of these (Ombrello haplotype 4 - associated with moderate ERAP1 activity) is found at higher frequency in UK Biobank CD patients when compared to either controls or UC (supplementary table 6). No difference in frequency of this haplotype was observed in the Southampton IBD cohort.

In both cohorts we found the *ERAP2* haplotypes identified by Raja and Kuiper. In Southampton cohort 3 of these haplotypes were seen: 2 of which resulting in decreased ERAP2 activity and 1 which resulted in normal ERAP2 activity. An equal proportion of individuals had a haplotype resulting in ERAP2 activity and inactivity in this cohort (45% each). In the UK Biobank cohort only 1 haplotype associated with decreased activity was identified. Neither cohort had differentially distributed *ERAP2* haplotypes.

Additional functional testing would be helpful to assess exactly how ERAP genes lead to UC pathogenesis. Functional testing of ERAP1 and ERAP2 enzymatic activity was conducted by Klunk et al. by culturing peripheral blood mononuclear cells (PBMCs) with killed and live strains of *Yersinia pestis* and with additional common pathogens i.e. *Salmonella, Listeria*, and influenza. This could be replicated with affected patients and controls to assess the size of the peptides being produced by ERAP1 and ERAP2 enzymes differ between groups using matrix-assisted laser desorption/ionization mass spectroscopy check the mass of peptide products. This would allow analysis if the peptides generated are the appropriate length for MHC-I presentation.

While difference in the distribution of variants with a higher predicted functional impact were identified between those with IBD and controls, between subtypes and for individuals with isolated UC and UC plus at least 1 other autoimmune disease, we were unable to clearly discern the direction of effect of these variants (protective or risk). This suggests that *ERAP* genes have a disease modifying effect and contributes to other genetic factors in the development of IBD and other common autoimmune diseases. While GenePy may be useful as part of polygenic risk score to account for the heterogenous rare variation in genes associated with IBD or autoimmune disease such as *ERAP1* and *ERAP2*, our data indicate the need for experimental data necessary to more clearly define how changes in these antigen-presenting proteins impact downstream cytokine signalling.

The bidirectional effect of variants in *ERAP* genes suggests multiple aetiological pathways could influence or modify susceptibility to UC and generalised autoimmunity. Increased enzymatic activity of ERAP1 and ERAP2 as selected for by the Black Death appears to have led to a hyperinflammatory response and self-reactivity (Klunk et al. 2022; Raja and Kuiper 2023); while reduced enzymatic activity of ERAP1 and ERAP2 has been implicated in hypoinflammation that hampers the immune response to infection, results in a reduction in pathogen clearance and activation of chronic inflammatory pathways (Yao et al. 2019). Variants in *NOD2*, an important IBD modulator, result in a similar hypoinflammatory response (Ashton et al. 2022). This hypoinflammation would likely be driven by rare variants and would not have undergone the same evolutionary pressure as common variants by the Black Death.

Our study was limited by lack of observation of non-coding variation that is largely not captured through exome sequencing. This limited the set of variants we observed that overlapped with SNPs previously associated with IBD and its subtypes identified in GWAS catalogue and OpenTargets. The Southampton IBD-cohort is an open study, and the duration of follow-up varies between patients. Identification of patients with multiple autoimmune disease is influenced by temporal opportunity to detect additional autoimmune diagnoses, although this limitation is independent of changes in *ERAP* genes and is unlikely to have influenced our observations.

Building on these data, further analysis is needed to uncover the precise functional consequences of variants within *ERAP* genes. Our data most consistently point to a significant modifying role of these genes in patients diagnosed with ulcerative colitis and those with multiple autoimmune diagnoses. Validation across deep and longitudinally phenotyped autoimmune patient cohorts will further advance our understanding of how difference in antigen presentation might represent an additional therapeutic target in combination therapies to modulate patient outcomes.

## Conclusions

The differences between the frequency of variants predicted to have functional effects between IBD subtypes and controls, between patients with UC and CD and those with an isolated IBD diagnosis and multiple autoimmune disease suggests a disease modifying effect of *ERAP* genes in UC and other common autoimmune diseases. Rare variants as a class are more frequent and confer greater functional consequence. Modelling the aggregated effects of all-frequency using GenePy implicates *ERAP* genes as modulators of susceptibility to UC and multiple concurrent autoimmune diagnoses. Our data evidence the need for experimental data clarifying the direction of impact of functional variation in downstream immune signalling. Such data, integrated with whole-gene burden approaches may be useful as part of a polygenic risk to guide clinical management in autoimmune disease.

## Supplementary Information

Below is the link to the electronic supplementary material.

Supplementary file1 (XLSX 509 KB)

## Data Availability

Southampton Genetics of IBD data are available on request only to protect individuals’ privacy. UK Biobank data access is managed the organisation.
